# Supplementary material for: CD73-mediated adenosine production by CD8 T cell-derived extracellular vesicles constitutes an intrinsic mechanism of immune suppression
Source: Nat Commun. 2021 Oct 8;12:5911. doi: 10.1038/s41467-021-26134-w (PMC8501027; doi:10.1038/s41467-021-26134-w)
Supplement: Supplementary file 1 — Supplementary Information [file 41467_2021_26134_MOESM1_ESM.pdf]

CD73-mediated adenosine production by CD8 T cell-derived extracellular vesicles constitutes an intrinsic mechanism of immune suppression

Supplementary Information

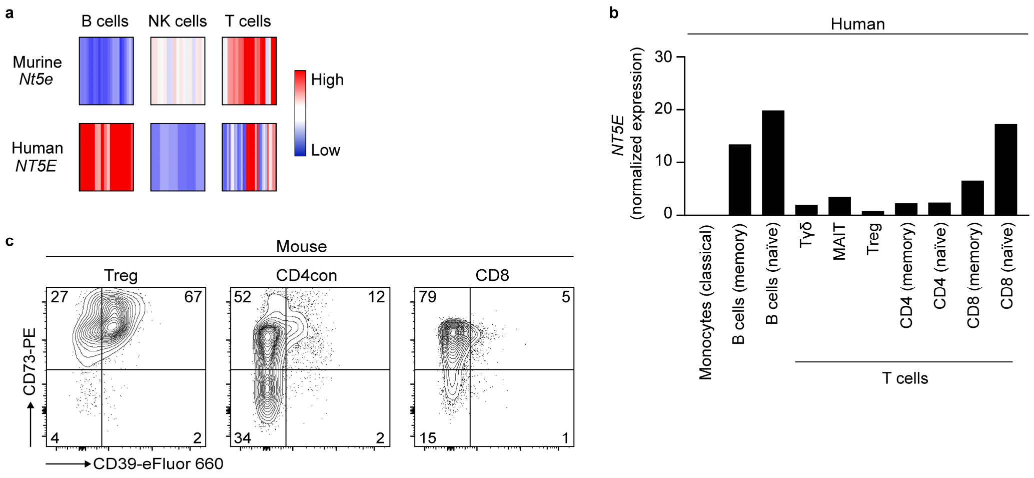

Supplementary Fig. 1 Human and murine T cell populations differ in the expression of CD39 and CD73. a Transcriptional profiles in matched B, NK and T cell subpopulations between mouse and human (data were obtained from the ImmGen database[1,2]). **b** Gene expression profile of *NT5E* (encoding CD73) in selected human immune cell populations (data obtained from the Human Protein Atlas[3,4]). **c** Representative dot plots of CD73 and CD39 expression on murine T cell subsets. Cells derived from the spleen of C57BL/6 mice.

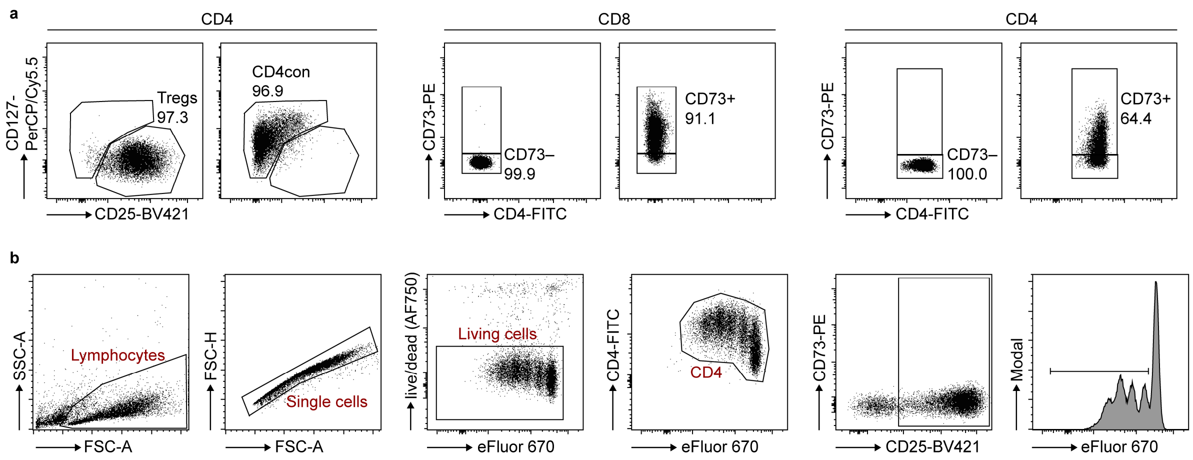

Supplementary Fig. 2 Purity of cell populations after cell sorting and gating strategy for T cell assays. a Representative dot plots showing the purity of sorted T cell populations. **b** Gating strategy to determine the activation and proliferation of activated CD4con T cells. Lymphocytes were defined by forward scatter (FSC) versus side scatter (SSC). Dead cells were excluded from the analysis. Expression of CD25 on $CD4^+$ cells was analyzed as a marker of activation, and $CD4^+$ cells showing eFluor 670 dye dilution were defined as proliferated cells.

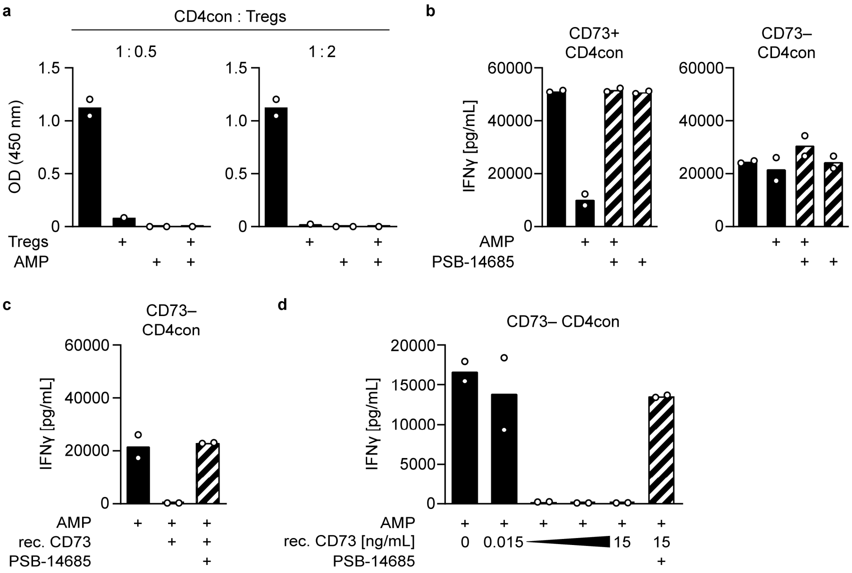

Supplementary Fig. 3 Adenosine-mediated suppression of conventional CD4 T cells is independent of Treg-derived CD73. Responder T cells were stimulated in the presence of the ADA inhibitor EHNA (10 µM). IFNγ production was determined by ELISA in the cell culture supernatants on day four. **a** CD4con T cells were stimulated and incubated with AMP (50 µM) and Tregs in the indicated ratio. **b** CD4con T cells were sorted into CD73⁻ and CD73⁺, the cells were incubated with AMP (50 µM) and the specific CD73 inhibitor PSB-14685 (10 µM). **c** CD4con CD73⁻ T cells were incubated with AMP (50 µM), the specific CD73 inhibitor PSB-14685 (10 µM) and soluble recombinant CD73 (15 ng/mL). **d** CD4con CD73⁻ T cells were incubated with AMP (50 µM) and different concentrations of soluble recombinant CD73 (three ten-fold serial dilutions starting with 15 ng/mL). PSB-14685 was used to block the highest concentration of recombinant CD73. Data are shown for one representative donor (mean of technical duplicates), all experiments were repeated at least three times.

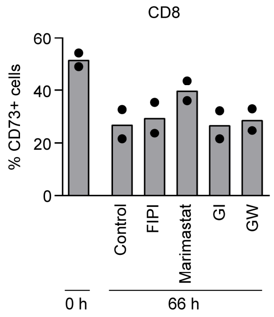

Supplementary Fig. 4 Inhibitors of phospholipase D and matrix metalloproteases do not prevent the loss of CD73 from the cell membrane. PBMCs were stimulated with αCD3 for 66 h. After 18 h, PLD inhibitor FIPI (20 µM), broad-spectrum MMP inhibitor Marimastat (50 µM), ADAM10 inhibitor GI254023X (GI, 3 µM), and ADAM10/17 inhibitor GW280264X (GW, 3 µM) were added to the cell culture. CD73 expression was determined by flow cytometry (gated on CD8 T cells). Data are shown for two donors (mean).

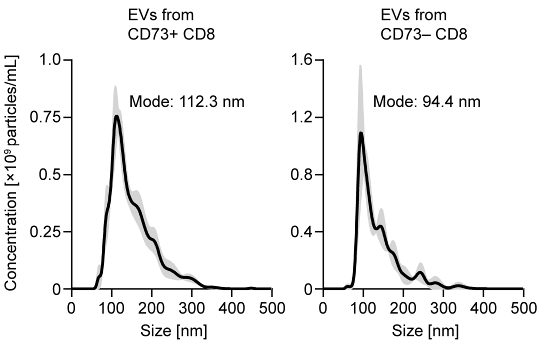

Supplementary Fig. 5 Cell culture supernatants of activated CD8 T cells contain extracellular vesicles. Size determination of EVs isolated from cell culture supernatants of activated $CD73^{+}$ and $CD73^{-}$ CD8 T cells (average of three measurements ± SEM).

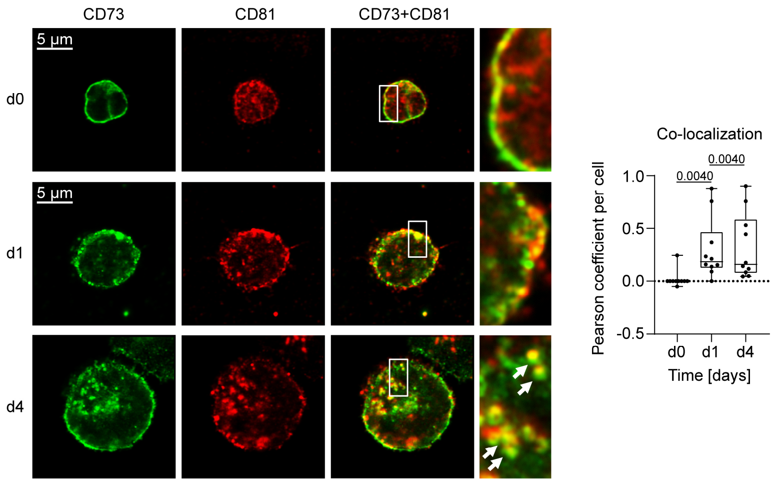

Supplementary Fig. 6 CD73 and tetraspanin CD81 co-localize after T cell activation. Microscopy analysis of CD73 and CD81 expression in CD8 T cells before and after activation. Pearson coefficient was determined to quantify the co-localization of CD73 and CD81. Data from ten high power fields were analyzed at each time point (center line: median, box limits: 25th to 75th percentiles, whiskers: min to max). Kruskal-Wallis test with Dunn's multiple comparisons test was used to compare Pearson coefficients of co-localization.

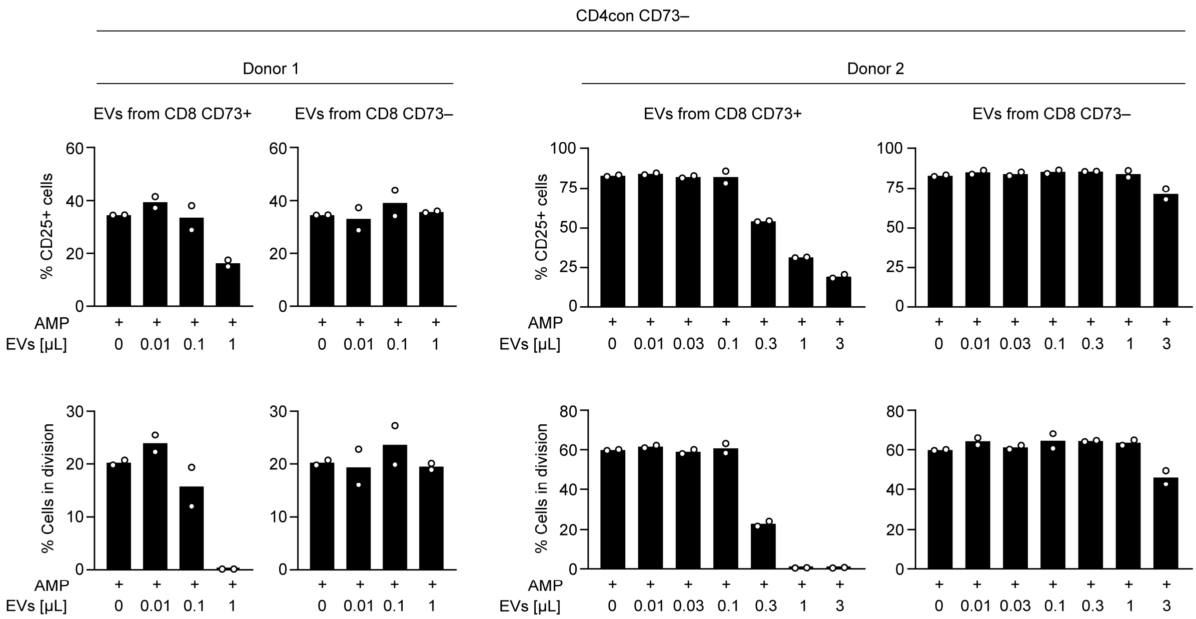

Supplementary Fig. 7 Extracellular vesicles derived from CD73⁺ CD8 T cells decrease the activation and proliferation of responder T cells in a dose-dependent manner. CD73⁻ CD4con T cells were stimulated with αCD3/αCD28 in the presence of the ADA inhibitor EHNA (10 µM) and incubated with AMP (50 µM) and EVs derived from CD73⁺ CD8 or CD73⁻ CD8 T cells as indicated. CD25 expression and proliferation were measured after four days by flow cytometry. Data are shown for two donors from independent experiments (mean of technical duplicates).

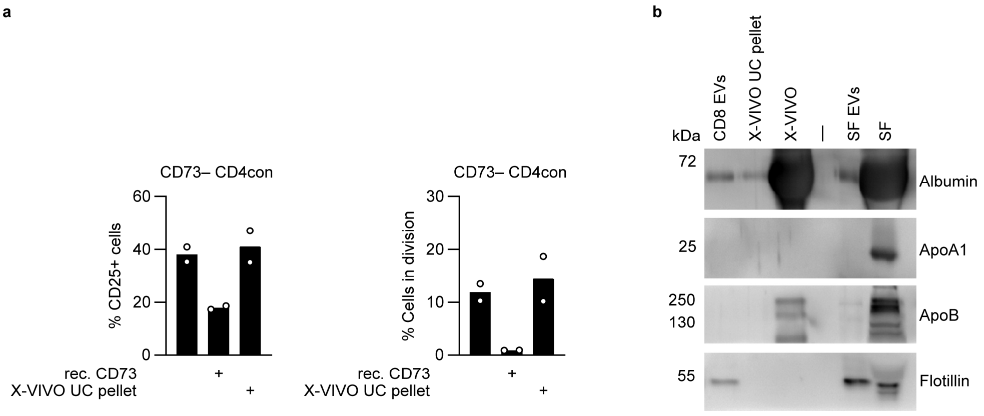

Supplementary Fig. 8 Assessment of contaminants in the extracellular vesicle preparations and effect on T cell function. a CD73⁻ CD4con T cells were stimulated with αCD3/αCD28 in the presence of the ADA inhibitor EHNA (10 µM) and incubated with AMP (50 µM), and recombinant CD73 (15 ng/mL) or pelleted material after ultracentrifugation of X-VIVO 15 medium (X-VIVO UC pellet). CD25 expression and proliferation were measured after four days by flow cytometry (mean of technical duplicates). UC: ultracentrifugation. **b** Western blot analysis of albumin and lipoproteins in EVs derived from T cell culture medium and SF of JIA patients. Samples loaded: 3.5 µg per EV sample, 15 µL of X-VIVO ultracentrifugation pellet (corresponding to 2 mL X-VIVO 15 medium), 15 µL X-VIVO 15 medium, 2 µL SF. Data are shown for one representative experiment out of two performed.

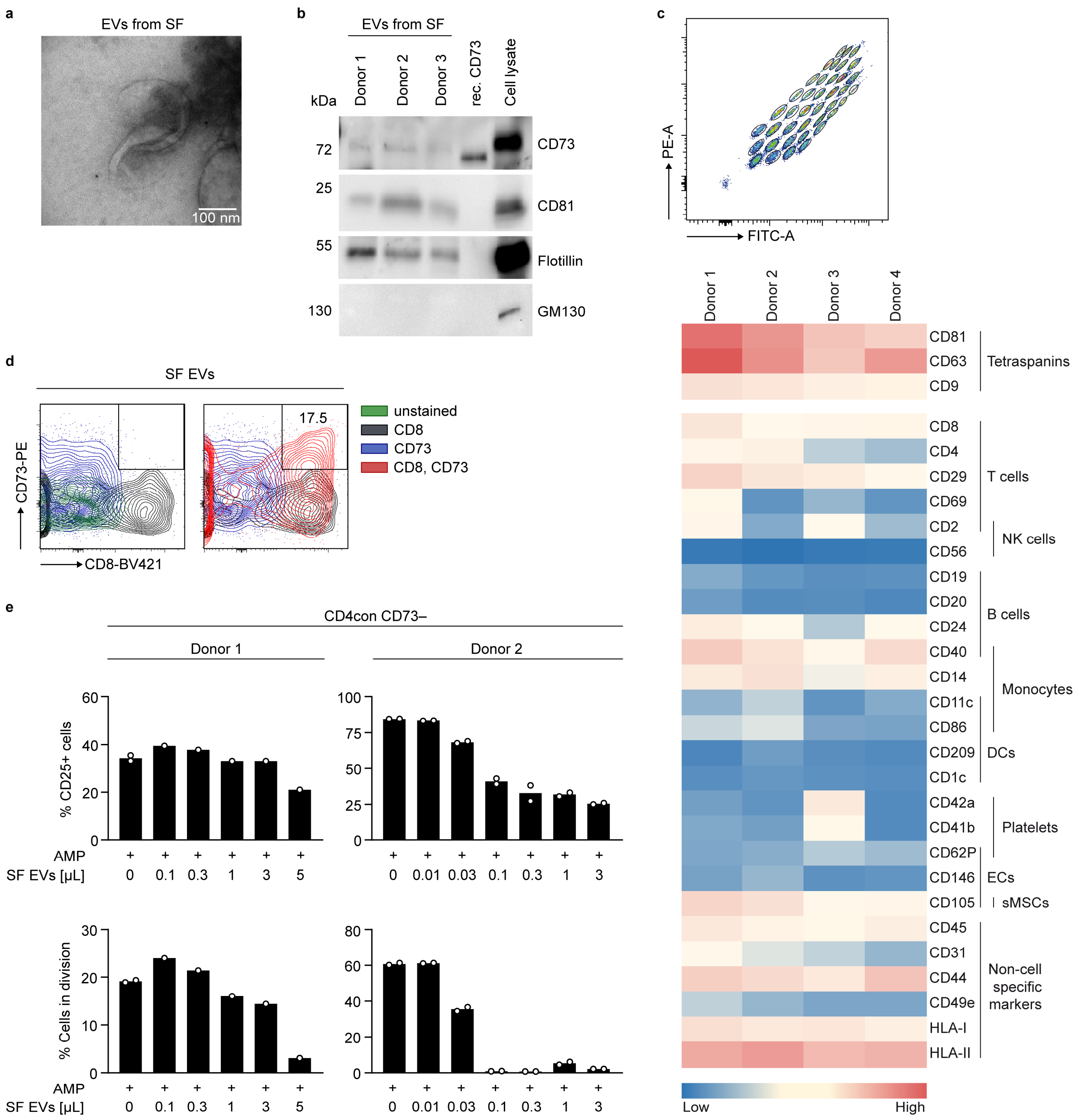

Supplementary Fig. 9 Characterization of synovial fluid-derived extracellular vesicles. a Representative electron microscopy image of EVs isolated from the SF of a patient with JIA (out of two patients analyzed). **b** Western blot analysis of CD73 and EV markers on EVs derived from the SF of patients with JIA. Cell lysate from stimulated CD8 T cells was used as positive control. Data are shown for three donors out of five analyzed in two independent experiments. **c** SF-derived EVs were captured by antibody-coated beads (dot plot shows the discrimination of bead populations by different fluorescence intensities, bead antigens are listed on the right side of the heat map), and detected with APC-labeled antibodies against tetraspanins CD9/CD63/CD81 by flow cytometry. The heat map shows background-corrected median fluorescence intensity of APC signals for the different bead populations for SF-derived EVs from four donors. DC: dendritic cells, EC: endothelial cells, sMSC: synovial mesenchymal stem cells. **d** Co-expression of CD8 and CD73 on SF-derived EVs detected by conventional flow cytometry. The gate for double-positive EVs was set based on FMO controls. **e** CD73⁻ CD4con T cells were

stimulated with αCD3/αCD28 in the presence of the ADA inhibitor EHNA (10 µM) and incubated with AMP (50 µM) and EVs derived from SF as indicated. CD25 expression and proliferation were measured after four days by flow cytometry. Data are shown for two donors from independent experiments (mean of technical duplicates).

Supplementary References

1. Heng, T. S. P. *et al.* The Immunological Genome Project: networks of gene expression in immune cells. *Nat. Immunol.* **9**, 1091–1094 (2008).
2. Shay, T. *et al.* Conservation and divergence in the transcriptional programs of the human and mouse immune systems. *Proc. Natl. Acad. Sci. U. S. A.* **110**, 2946–2951 (2013).
3. Uhlen, M. *et al.* A genome-wide transcriptomic analysis of protein-coding genes in human blood cells. *Science.* **366**, eaax9198 (2019).
4. Blood atlas - NT5E - The Human Protein Atlas. Available at: https://www.proteinatlas.org/ENSG00000135318-NT5E/blood. (Accessed: May 7, 2021)
