# Supplementary material for: CD73-mediated adenosine production by CD8 T cell-derived extracellular vesicles constitutes an intrinsic mechanism of immune suppression
Source: Nat Commun. 2021 Oct 8;12:5911. doi: 10.1038/s41467-021-26134-w (PMC8501027; doi:10.1038/s41467-021-26134-w)
Supplement: Supplementary file 3 — Reporting summary [file 41467_2021_26134_MOESM3_ESM.pdf]

nature research

Corresponding author(s): Riekje Winzer/Eva Tolosa

Last updated by author(s): Aug 30, 2021

Reporting Summary

Nature Research wishes to improve the reproducibility of the work that we publish. This form provides structure for consistency and transparency in reporting. For further information on Nature Research policies, see our Editorial Policies and the Editorial Policy Checklist.

Statistics

For all statistical analyses, confirm that the following items are present in the figure legend, table legend, main text, or Methods section.

n/a	Confirmed	
[ ]	[x]	The exact sample size (*n*) for each experimental group/condition, given as a discrete number and unit of measurement
[ ]	[x]	A statement on whether measurements were taken from distinct samples or whether the same sample was measured repeatedly
[ ]	[x]	The statistical test(s) used AND whether they are one- or two-sided<br>*Only common tests should be described solely by name; describe more complex techniques in the Methods section.*
[x]	[ ]	A description of all covariates tested
[ ]	[x]	A description of any assumptions or corrections, such as tests of normality and adjustment for multiple comparisons
[ ]	[x]	A full description of the statistical parameters including central tendency (e.g. means) or other basic estimates (e.g. regression coefficient) AND variation (e.g. standard deviation) or associated estimates of uncertainty (e.g. confidence intervals)
[ ]	[x]	For null hypothesis testing, the test statistic (e.g. *F*, *t*, *r*) with confidence intervals, effect sizes, degrees of freedom and *P* value noted<br>*Give P values as exact values whenever suitable.*
[x]	[ ]	For Bayesian analysis, information on the choice of priors and Markov chain Monte Carlo settings
[x]	[ ]	For hierarchical and complex designs, identification of the appropriate level for tests and full reporting of outcomes
[ ]	[x]	Estimates of effect sizes (e.g. Cohen's *d*, Pearson's *r*), indicating how they were calculated

Our web collection on statistics for biologists contains articles on many of the points above.

Software and code

Policy information about availability of computer code

Data collection	FACSDiva v8.0-v9.0 (BD), ChemStation C.01.05 (Agilent Technologies), Wallac 1420 Manager 3.0 (PerkinElmer), NTA 3.0 (Malvern Instruments), Quantity One 4.6.6 (Bio-Rad), ZENblue 2.6 (ZEISS)
Data analysis	FlowJo v10.2-v10.7 (BD), Prism 8 (GraphPad), ChemStation C.01.05 (Agilent Technologies)

For manuscripts utilizing custom algorithms or software that are central to the research but not yet described in published literature, software must be made available to editors and reviewers. We strongly encourage code deposition in a community repository (e.g. GitHub). See the Nature Research guidelines for submitting code & software for further information.

Data

Policy information about availability of data

All manuscripts must include a data availability statement. This statement should provide the following information, where applicable:

- Accession codes, unique identifiers, or web links for publicly available datasets
- A list of figures that have associated raw data
- A description of any restrictions on data availability

The authors declare that the data supporting the findings of this study are available within the paper and its supplementary information files, or in the source data file. Data from publicly available sources shown in this paper can be obtained from the Human Protein Atlas (https://www.proteinatlas.org/ENSG00000135318-NT5E/blood, NT5E expression in human blood). Source data are provided with this paper.

Field-specific reporting

Please select the one below that is the best fit for your research. If you are not sure, read the appropriate sections before making your selection.

☒ Life sciences ☐ Behavioural & social sciences ☐ Ecological, evolutionary & environmental sciences

For a reference copy of the document with all sections, see nature.com/documents/nr-reporting-summary-flat.pdf

Life sciences study design

All studies must disclose on these points even when the disclosure is negative.

Sample size	No sample-size calculation was performed. Sample sizes were based on common standards in the field. All experiments were performed at least three times, the exact number is written in the figure legends.
Data exclusions	Data was only excluded on the basis of very clear technical failure.
Replication	All the experiments were repeated at least three times in independent approaches unless stated otherwise in the manuscript. All attempts at replication were successful.
Randomization	There was no need for subject randomization because there are no experimental groups in this study.
Blinding	Blinding was not relevant in this study, because cells from each donor were treated with the same compounds and there was no group allocation of the donors.

Reporting for specific materials, systems and methods

We require information from authors about some types of materials, experimental systems and methods used in many studies. Here, indicate whether each material, system or method listed is relevant to your study. If you are not sure if a list item applies to your research, read the appropriate section before selecting a response.

Materials & experimental systems

n/a	Involved in the study	
☐	☒	Antibodies
☒	☐	Eukaryotic cell lines
☒	☐	Palaeontology and archaeology
☐	☒	Animals and other organisms
☐	☒	Human research participants
☒	☐	Clinical data
☒	☐	Dual use research of concern

Methods

n/a	Involved in the study	
☒	☐	ChIP-seq
☐	☒	Flow cytometry
☒	☐	MRI-based neuroimaging

Antibodies

Antibodies used

Flow cytometry
Anti-human antibodies:
CD3-PerCPCy5.5 (BioLegend, #317336, clone OKT3)
CD3-PerCPCy5.5 (eBioscience, #45003742, clone OKT3)
CD3-APC (BioLegend, #300412, clone UCHT-1)
CD3-BV785 (BioLegend, #317330, clone OKT3)
CD3-BV510 (BioLegend, #317332, clone OKT3)
CD4-APC (BioLegend, #344613, clone SK3)
CD4-FITC (BioLegend, #300506, clone RPA-T4)
CD4-AF488 (BioLegend, #300519, clone RPA-T4)
CD4-V500 (BD Biosciences, #560768, clone RPA-T4)
CD4-AF700 (BioLegend, #317426, clone OKT4)
CD8α-BV510 (BioLegend, #301048, clone RPA-T8)
CD8α-BV605 (BioLegend, #301040, clone RPA-T8)
CD8α-FITC (BioLegend, #301006, clone RPA-T8)
CD8α-BV421 (BioLegend, #301036, clone RPA-T8)
CD8α-AF700 (BioLegend, #300920, clone HIT8a)
CD9-APC (BioLegend, #312107, clone HI9a)
CD14-V450 (BD Biosciences, #560349, clone M5E2)
CD16-APCCy7 (BioLegend, #302018, clone 3G8)

CD19-PECy7 (BioLegend, #302216, clone HIB19)
CD25-PE (BD Biosciences, #555432, clone 2A3)
CD25-BV421 (BioLegend, #302630, clone BC96)
CD39-PECy7 (BioLegend, #328212, clone A1)
CD45-V500 (BD Biosciences, #560777, clone HI30)
CD56-APC (Beckman Coulter, #IM2474, clone N901)
CD73-PE (BioLegend, #344004, clone AD2)
CD73-APC (BioLegend, #344006, clone AD2)
CD127-PerCPCy5.5 (BioLegend, #351322, clone A019D5)
CD127-BV650 (BioLegend, #351326, A019D5)
TCRγδ-PE (BD Biosciences, #333141, clone 11F2)
MACSPlex Exosome Kit (Miltenyi, #130-122-209)

Anti-mouse antibodies:
CD3-BV421 (BioLegend, #100228, clone 17A2)
CD4-FITC (BioLegend, #100510, clone RM4-5)
CD25-BV510 (BioLegend, #102042, clone PC61)
CD73-PE (BioLegend, #127205, clone TY/11.8)
CD8-PECy7 (eBioscience, #25-0081-82, clone 53-6.7)
CD19-PerCPCy5.5 (eBioscience, #45-1293-80, clone 1D3)
CD39-eFluor 660 (eBioscience, #50-0391-80, clone 24DMS1)

Western blot
Anti-human CD73 (Cell Signaling, #13160, clone D7F9A)
Anti-human CD81 (Cell Signaling, #56039, clone D3N2D)
Anti-human Flotillin-1 (BD Biosciences, #610820, clone 18/Flotillin-1)
Anti-human GM130 (BD Biosciences, #610822, clone 35/GM130)
Anti-human Albumin (Santa Cruz, #sc-271605, clone F-10)
Anti-human apoA1 (Santa Cruz, #sc-23605, clone E-20)
Anti-human apoB (Acris, #R1032P, polyclonal)
Anti-rabbit-HRP-conjugated secondary antibody (Cell Signaling, #7074)
Anti-mouse-HRP-conjugated secondary antibody (Cell Signaling, #7076)
Anti-goat-HRP-conjugated secondary antibody (Jackson ImmunoResearch Laboratories, #205-035-108)

Confocal fluorescence microscopy
Anti-human CD73 unconjugated (BioLegend, #344002, clone AD2)
Anti-mouse IgG-Cy2-conjugated secondary antibody (Jackson ImmunoResearch Laboratories, #715-225-150)
Anti-human CD9-APC (BioLegend, #312107, clone HI9a)
Anti-human CD81-APC (BioLegend, #349509, clone 5A6)

Electron microscopy
Anti-human CD73 unconjugated (BioLegend, #344002, clone AD2)
Anti-mouse IgG-Gold-conjugated secondary antibody (Sigma, #G7777)

Different lots of each antibody were used for the experiments shown in the paper.

Validation

All antibodies are commercially available and datasheets as well as product citations can be obtained from the manufacturer's website. The companies use several approaches to validate their antibodies: BioLegend tests the specificity of their antibodies on one to three target cell types including positive and negative cell types and each lot undergoes quality control testing (https://www.biolegend.com/en-us/quality/quality-control). BD Biosciences confirms the specificity of their flow cytometry reagents with multiple applications and titrates them on positive or negative cells (https://www.bdbiosciences.com/en-eu/products/reagents/flow-cytometry-reagents/research-reagents/quality-and-reproducibility). eBioscience/Thermo Fisher Scientific performs a two-step testing approach including a target specificity verification and functional application validation (https://www.thermofisher.com/de/de/home/life-science/antibodies/invitrogen-antibody-validation.html). Cell Signaling uses different validation steps to ensure antibody specificity, including e.g. siRNA knock-down, analysis of multiple cell lines and activator treatment (https://www.cellsignal.com/about-us/our-approach-process/antibody-validation-western-blotting).
We routinely titrate all antibodies in our lab.

Following antibodies were validated/quality tested for flow cytometry application, as indicated on the manufacturer's websites.
Anti-human antibodies:
CD3-PerCPCy5.5: https://www.biolegend.com/en-us/products/percp-cyanine5-5-anti-human-cd3-antibody-8220
CD3-PerCPCy5.5: https://www.fishersci.com/shop/products/anti-h-cd3-percp-cy55-100-ts/5015744
CD3-APC: https://www.biolegend.com/en-us/products/apc-anti-human-cd3-antibody-861
CD3-BV785: https://www.biolegend.com/en-us/search-results/brilliant-violet-785-anti-human-cd3-antibody-7977
CD3-BV510: https://www.biolegend.com/en-us/products/brilliant-violet-510-anti-human-cd3-antibody-8009
CD4-APC: https://www.biolegend.com/en-us/products/apc-anti-human-cd4-antibody-6378
CD4-FITC: https://www.biolegend.com/en-us/products/fitc-anti-human-cd4-antibody-825
CD4-AF488: https://www.biolegend.com/en-us/products/alexa-fluor-488-anti-human-cd4-antibody-2727

CD4-V500: https://www.bdbiosciences.com/en-us/products/reagents/flow-cytometry-reagents/research-reagents/single-color-antibodies-ruo/v500-mouse-anti-human-cd4.560768
CD4-AF700: https://www.biolegend.com/en-us/products/alexa-fluor-700-anti-human-cd4-antibody-3661
CD8α-BV510: https://www.biolegend.com/en-us/products/brilliant-violet-510-anti-human-cd8a-antibody-8000
CD8α-BV605: https://www.biolegend.com/en-us/products/brilliant-violet-605-anti-human-cd8a-antibody-7651
CD8α-FITC: https://www.biolegend.com/en-us/products/fitc-anti-human-cd8a-antibody-834
CD8α-BV421: https://www.biolegend.com/en-us/products/brilliant-violet-421-anti-human-cd8a-antibody-7152
CD8α-AF700: https://www.biolegend.com/en-us/products/alexa-fluor-700-anti-human-cd8a-antibody-3434
CD9-APC: https://www.biolegend.com/en-us/products/apc-anti-human-cd9-antibody-15072
CD14-V450: https://www.bdbiosciences.com/en-de/products/reagents/flow-cytometry-reagents/research-reagents/single-color-antibodies-ruo/v450-mouse-anti-human-cd14.560349
CD16-APCCy7: https://www.biolegend.com/en-us/products/apc-cyanine7-anti-human-cd16-antibody-1904
CD19-PECy7: https://www.biolegend.com/en-us/products/pe-cyanine7-anti-human-cd19-antibody-1911
CD25-PE: https://www.bdbiosciences.com/en-de/products/reagents/flow-cytometry-reagents/research-reagents/single-color-antibodies-ruo/pe-mouse-anti-human-cd25.555432
CD25-BV421: https://www.biolegend.com/en-us/products/brilliant-violet-421-anti-human-cd25-antibody-7139
CD39-PECy7: https://www.biolegend.com/en-us/products/pe-cyanine7-anti-human-cd39-antibody-6320
CD45-V500: https://www.bdbiosciences.com/en-de/products/reagents/flow-cytometry-reagents/research-reagents/single-color-antibodies-ruo/v500-mouse-anti-human-cd45.560777
CD56-APC: https://www.beckman.de/reagents/coulter-flow-cytometry/antibodies-and-kits/single-color-antibodies/cd56/im2474?country=DE&_ga=2.63741992.600523261.1629272335-462946919.1629272335
CD73-PE: https://www.biolegend.com/en-us/products/pe-anti-human-cd73-ecto-5-nucleotidase-antibody-6092
CD73-APC: https://www.biolegend.com/en-us/products/apc-anti-human-cd73-ecto-5-nucleotidase-antibody-6093
CD127-PerCPCy5.5: https://www.biolegend.com/en-us/products/percp-cyanine5-5-anti-human-cd127-il-7ralpha-antibody-7217
CD127-BV650: https://www.biolegend.com/en-us/products/brilliant-violet-650-anti-human-cd127-il-7ralpha-antibody-7673
TCRγδ-PE: https://www.bdbiosciences.com/en-de/products/reagents/flow-cytometry-reagents/clinical-diagnostics/single-color-antibodies-asr-ivd-ce-ivd/anti-tcr-pe.333141#

Anti-mouse antibodies:
CD3-BV421: https://www.biolegend.com/en-us/products/brilliant-violet-421-anti-mouse-cd3-antibody-7326
CD4-FITC: https://www.biolegend.com/en-us/products/fitc-anti-mouse-cd4-antibody-480
CD25-BV510: https://www.biolegend.com/en-us/products/brilliant-violet-510-anti-mouse-cd25-antibody-8663
CD73-PE: https://www.biolegend.com/en-us/products/pe-anti-mouse-cd73-antibody-4681
CD8-PECy7: https://www.thermofisher.com/antibody/product/CD8a-Antibody-clone-53-6-7-Monoclonal/25-0081-82
CD19-PerCPCy5.5: https://www.thermofisher.com/antibody/product/CD19-Antibody-clone-eBio1D3-1D3-Monoclonal/45-0193-82
CD39-eFluor 660: https://www.thermofisher.com/antibody/product/CD39-Antibody-clone-24DMS1-Monoclonal/50-0391-82

Following antibodies were validated/quality tested for western blot application, as indicated on the manufacturer's websites.
Anti-human CD73: https://www.cellsignal.com/products/primary-antibodies/nt5e-cd73-d7f9a-rabbit-mab/13160
Anti-human CD81: https://www.cellsignal.com/products/primary-antibodies/cd81-d3n2d-rabbit-mab/56039?site-search-type=Products&N=4294956287&Ntt=%2356039&fromPage=plp&_requestid=4093757
Anti-human Flotillin-1: https://www.bdbiosciences.com/en-ca/products/reagents/microscopy-imaging-reagents/immunofluorescence-reagents/purified-mouse-anti-flotillin-1.610820
Anti-human GM130: https://www.bdbiosciences.com/en-ca/products/reagents/microscopy-imaging-reagents/immunofluorescence-reagents/purified-mouse-anti-gm130.610822
Anti-human Albumin: https://www.scbt.com/p/alb-antibody-f-10?productCanUrl=alb-antibody-f-10&_requestid=906066
Anti-human apoA1: https://www.scbt.com/p/apoa-i-antibody-e-20?requestFrom=search
Anti-human apoB: https://www.origene.com/catalog/antibodies/primary-antibodies/r1032p/apolipoprotein-b-apob-goat-polyclonal-antibody
Anti-rabbit-HRP-conjugated secondary antibody: https://www.cellsignal.com/products/secondary-antibodies/anti-rabbit-igg-hrp-linked-antibody/7074
Anti-mouse-HRP-conjugated secondary antibody: https://www.cellsignal.com/products/secondary-antibodies/anti-mouse-igg-hrp-linked-antibody/7076
Anti-goat-HRP-conjugated secondary antibody: https://www.jacksonimmuno.com/catalog/products/205-035-108

Following antibodies were validated/quality tested for fluorescence microscopy.
Anti-mouse IgG-Cy2-conjugated secondary antibody: https://www.jacksonimmuno.com/catalog/products/715-225-150
Anti-human CD73 unconjugated (https://www.biolegend.com/en-us/products/purified-anti-human-cd73-ecto-5-nucleotidase-antibody-6091) was validated for fluorescence microscopy by using CD73pos and CD73neg samples
Anti-human CD9-APC: https://www.biolegend.com/en-us/products/apc-anti-human-cd9-antibody-15072
Anti-human CD81-APC: https://www.biolegend.com/en-us/products/apc-anti-human-cd81-tapa-1-antibody-10228

Following antibodies were validated/quality tested for electron microscopy.
Anti-mouse IgG-Gold-conjugated secondary antibody: https://www.sigmaaldrich.com/DE/en/product/sigma/g7777
Anti-human CD73 unconjugated (https://www.biolegend.com/en-us/products/purified-anti-human-cd73-ecto-5-nucleotidase-antibody-6091) was validated for electron microscopy by using CD73pos and CD73neg samples

Animals and other organisms

Policy information about studies involving animals; ARRIVE guidelines recommended for reporting animal research

Laboratory animals	Only Supplementary Fig. 1c involves animals (n = 3): C57BL/6, female, 12 weeks. Mice were housed under specific-pathogen-free conditions with standard food and water ad libitum in a 12h light / 12h dark cycle. Humidity and ambient temperature were maintained between 45-65% and 20-24°C, respectively.
Wild animals	The study did not involve wild animals.
Field-collected samples	The study did not involve samples collected from the field.
Ethics oversight	Hamburg Authority for Health and Consumer Protection, Veterinary Affairs/Food Safety, protocol ORG983.

Note that full information on the approval of the study protocol must also be provided in the manuscript.

Human research participants

Policy information about studies involving human research participants

Population characteristics	The study population were adults of both sexes (age 18 - 65), defined as healthy (no diagnosis which excludes them from blood donation at the University Medical Center Hamburg-Eppendorf (UKE)). The JIA donors were children (age under 18) of both sexes, diagnosed with oligo- or polyarthritis. The samples from JIA patients were obtained during disease relapse and before acute steroidal treatment. Some of the patients were receiving disease-modifying anti-rheumatic drugs (DMARDs) or biologicals. All donors were anonymized for these analyses. No genotypic information was obtained.
Recruitment	Buffy coats were obtained from the blood bank of the University Medical Center Hamburg-Eppendorf (UKE) and randomly assigned to us. Peripheral blood was drawn from healthy volunteers visiting the UKE. Blood and synovial fluid (SF) of patients with juvenile idiopathic arthritis (JIA) were obtained from children presenting to the outpatient clinic of the UKE, the Altona Children's Hospital, the University Hospital Schleswig-Holstein (campus Lübeck), or the Medical Center Bad Bramstedt, due to exacerbation of disease or first appearance of symptoms.
Ethics oversight	All samples were handled according to corresponding ethics protocols (Ethics Committee of the Hamburg Chamber of Physicians, protocols PV5139 for samples from healthy donors, and PV3746 for samples from JIA patients) and informed consent was obtained from all donors.

Note that full information on the approval of the study protocol must also be provided in the manuscript.

Flow Cytometry

Plots

Confirm that:

- [x] The axis labels state the marker and fluorochrome used (e.g. CD4-FITC).
- [x] The axis scales are clearly visible. Include numbers along axes only for bottom left plot of group (a 'group' is an analysis of identical markers).
- [x] All plots are contour plots with outliers or pseudocolor plots.
- [x] A numerical value for number of cells or percentage (with statistics) is provided.

Methodology

Sample preparation	Human mononuclear cells were obtained by density gradient centrifugation from buffy coats, peripheral blood of healthy donors and patients with juvenile idiopathic arthritis (JIA), and from the synovial fluid of JIA patients. Single-cell suspensions of murine splenocytes were prepared by processing the spleen through a 70 µm strainer. Human mononuclear cells or T cells and murine splenocytes were pre-incubated with immunoglobulins to block unspecific binding, and stained with fluorescence-labeled antibodies for 30 min at 4°C. For dead cell exclusion, a live/dead dye (Thermo Fisher Scientific) was included. The staining cocktails were designed to minimize the effects of spectral overlap. Prior to analysis, a compensation matrix was calculated after single color staining of human PBMCs.
Instrument	FACSCanto II, FACSCelesta, LSR Fortessa, FACSAria IIIU (all BD Biosciences).
Software	FACSDiva was used for data collection and FlowJo for data analysis (both BD).
Cell population abundance	Cells were sorted with a FACSAria IIIU sorter with settings for high purity (4-way-purity sort). The purity of each sorted cell populations was determined by flow cytometry, and was always greater than 95% for Tregs and CD4con, and higher than 99% for CD73 negative CD8 and CD4 T cells. For CD73 positive T cells, highest purity was not essential for the assays, and the sorting gates were therefore less stringent (purity of cell populations is shown in Supplementary Fig. 2a).
Gating strategy	FSC-A and SSC-A were used to define the starting population, and the 'lymphocyte' gate was adapted for ex-vivo or in vitro cell analysis (stimulated cells). FSC-A/FSC-H and SSC-A/SSC-H plots were used to exclude doublets. A dead cell exclusion dye (amine-reacting dye in the R-780 channel) was used to gate living cells.

Depending on the experiment, following gating strategies were applied:
-Expression of ectonucleotidases on T cell subsets: T cells were gated on the basis of CD3 expression; CD8 T cells were defined as CD8highCD4neg; Tregs were defined as CD4pos, CD25high, CD127neg; CD4con T cells were defined as non-Treg CD4pos T cells (gating strategy is shown in the source data file to Fig. 1e and Supplementary Fig. 1c for human and murine samples, respectively).
-T cell assays: CD25pos and proliferating cells (defined by eFluor 670 dilution) were gated on CD4pos cells (after excluding Tregs identified as described above). Activation and proliferation gates were set based on non-activated cells from the same donor (gating strategy is shown in Supplementary Fig. 2b).
-T cell suppression assays (co-culture of CD4con and Tregs): Activated cells (CD25pos) and proliferating cells (defined by eFluor 670 dilution) were gated on CD4pos, eFluor 670pos/low cells (exclusion of Tregs which are eFluor 670neg); gates were set based on non-activated cells from the same donor (gating strategy is shown in the source data file to Fig. 1g).
-Phenotyping of peripheral blood and synovial fluid of JIA patients: B and T cells were gated out of CD45pos mononuclear cells by CD19 and CD3 expression, respectively; CD3pos cells were further separated into CD4 T cells (CD4pos), Tgd cells (gdTCRpos) and CD8 T cells (CD4neg, gdTCRneg); NK cells were gated out of CD19neg, CD3neg cells by CD56 expression; CD56neg cells were further used for gating monocytes (CD14pos and/or CD16pos) (gating strategy is shown in the source data file to Fig. 7a).

☒ Tick this box to confirm that a figure exemplifying the gating strategy is provided in the Supplementary Information.
